# MicroRNA-21 Mediates the Inhibiting Effect of Praziquantel on NLRP3 Inflammasome in *Schistosoma japonicum* Infection

**DOI:** 10.3389/fvets.2019.00517

**Published:** 2020-02-12

**Authors:** Delong Kong, Hongfei Guo, Zhongkui Lu, Jie Cui

**Affiliations:** ^1^Jiangsu Key Laboratory of Immunity and Metabolism, Laboratory of Infection and Immunity, Department of Pathogenic Biology and Immunology, Xuzhou Medical University, Xuzhou, China; ^2^Key Laboratory of Pathogen Biology of Jiangsu Province, Department of Pathogen Biology, Nanjing Medical University, Nanjing, China; ^3^Department of Physiology, Xuzhou Medical University, Xuzhou, China

**Keywords:** praziquantel, NLRP3 inflammasome, miR-21, schistosomiasis, macrophages

## Abstract

Praziquantel (PZQ), a traditional helminthicide drug, has been shown to exert an anti-inflammatory effect on splenomegaly induced by schistosomiasis via regulating macrophage polarization. Meanwhile, miR-21 has been demonstrated to control macrophage polarization. However, the role of miR-21 in the regulation of macrophage polarization by PZQ in schistosomiasis is still unclear. In the present study, we found that M1-type macrophages were the predominant splenic macrophages in chronic schistosomiasis and that NLRP3 inflammasome–related molecules were upregulated. PZQ inhibited NLRP3 inflammasome in M1 macrophages and reduced the expression of miR-21. Furthermore, using the methods of quantitative real-time PCR and transfection, the downregulation of NLRP3/IL-1β by PZQ in M1 macrophages were reversed by miR-21 overexpression. These results indicated that miR-21 was involved in the inhibiting effect of PZQ on activation of NLRP3 inflammasome. Moreover, miR-21 might target Smad7 to mediate the anti-inflammatory effect of PZQ in polarized macrophages. This study provides an in-depth mechanism of PZQ in the treatment of schistosomiasis.

## Introduction

Schistosomiasis is a widespread zoonotic parasitic disease (1). So far, more than 200 million people have been infected with schistosomiasis worldwide, with 120 million showing clinical symptoms (2). China is one of the countries with the highest prevalence of *Schistosoma japonicum* (*S. japonicum*) infection. Humans with chronic *S. japonicum* infections often develop into schistosomiasis characterized by hepatosplenomegaly, portal hypertension, and so on (3). Therefore, in the case of *S. japonicum* infections, splenomegaly and hypersplenism have recently drawn a great deal of interest.

Macrophages are the essential components of immunity in the spleen (4). Studies have shown that splenic macrophages exhibit enhanced phagocytic ability and cytokine secretion in hypersplenism (5, 6). In response to various stimuli, macrophages may undergo classical (M1) macrophage-activation or, alternatively, M2 macrophage-activation. M1 macrophages are associated with inflammation in the host defense and antitumor immunity, while M2 macrophages dampen the inflammatory process by secreting anti-inflammatory cytokines (5, 6). It is reported that pro-inflammatory cytokines are upregulated significantly in splenic macrophages in cirrhotic patients with hypersplenism (7). Meanwhile, pro-inflammatory cytokines produced by M1 macrophages contribute to the pathological damage of chronic venous leg ulcers (8). IL-1β plays a critical role as a potent pro-inflammatory cytokine in infectious diseases, autoimmune diseases, and aseptic inflammation (9, 10). IL-1β production mainly depends on the activation of NLRP3 inflammasome (11). NLRP3 inflammasome is a multiprotein complex, which plays a critical role in innate immunity by participating in the activation of caspase-1 and production of IL-1β and IL-18 (12, 13). It has been reported that NLRP3 inflammasome is mainly activated in M1 macrophages but not in M2 macrophages (14). Therefore, the activation of NLRP3 inflammasome in M1 macrophages plays an important role in the response to infection and the pathogenesis of tissue insult.

Praziquantel (PZQ) is well-known for its schistosomicidal effect as a traditional anti-schistosomiasis drug (15). Our previous studies have revealed that long-term PZQ treatment had anti-inflammatory effects and considerably improved *S. japonicum*-induced splenomegaly and liver fibrosis (16, 17). Moreover, PZQ alleviated the pathological damage of the spleen in mice with chronic *S. japonicum* infection by regulating macrophage polarization and attenuating the phagocytic activity of M1 macrophages (18). However, little is known about the underlying mechanisms of anti-inflammatory effects of PZQ. Moreover, the roles of PZQ in macrophages polarization remain elusive.

MicroRNAs (miRs) are endogenous, single-stranded, non-coding small RNAs with the principal function of inhibiting gene expression at the transcriptional level (19). It is reported that miR-21 is involved in the occurrence and progress of liver inflammation and fibrosis (20, 21). miR-21 could inhibit Spry1 by enhancing ERK kinase activity in cardiac fibroblasts and hepatic astrocytes (22). In addition, miR-21 influences the activation of NLRP3 inflammasome–related factors by regulating the expression of the Smad7 protein (23). Moreover, miR-21 inhibition impairs expression of M2 signature genes but not M1 genes, indicating that miR-21 is involved in homeostatic macrophage polarization (24). However, whether miR-21 is involved in the process of inhibiting inflammatory response and regulating macrophages polarization by PZQ is unclear.

Considering the traditional use of PZQ as an anti-parasitic drug against schistosomiasis and other helminthiases, as well as immunomodulatory function of PZQ by our previous data, we aim to evaluate whether miR-21 is involved in the effect of PZQ on NLRP3 inflammatory response. Findings from the current study will hopefully stimulate further investigations on the mechanism of PZQ's effect on pathological damage of the spleen caused by schistosomiasis.

## Materials and Methods

### Mouse Model of Chronic Schistosomiasis

Six-week-old female C57BL/6 mice were purchased from the Animal Core Facility of Nanjing Medical University, Nanjing, China. The mice were fed in a specific pathogen-free microenvironment before being infected. For infections, mice were exposed to 14 ± 2 *S. japonicum* cercariae percutaneously and fed for 12 weeks post-infection. Cercariae were obtained from the Jiangsu Parasitology Institute, Wuxi, China.

### Enzyme-Linked Immunosorbent Assay

Total cytokines were taken from cell culture medium, and the secretion of IL-1β was detected using a commercially available enzyme-linked immunosorbent assay (ELISA) kit according to the manufacturer's instructions (eBioscience, USA).

### Histology Assays

The spleen tissues were fixed in a neutral buffered formalin solution and then embedded in paraffin. Sections (6 μm thick) of splenic slices were stained with hematoxylin–eosin (H&E) to identify the inflammation and necrosis under light microscopy.

### Cell Isolation, Culture, Plasmid Construction, and Transfection

The mice were sacrificed after 2% pentobarbital sodium anesthesia. The isolation of splenic macrophage has been described previously (25). Briefly, splenocytes were separated by pressing the spleens followed by cracking the erythrocytes. After incubation in Dulbecco modified Eagle medium (DMEM) for 2 h with 10% fetal bovine serum (37°C, 95% humidity, 5% CO_2_), adherent splenic macrophages were harvested and positively sorted as CD11b^+^ cells by using CD11b microbeads following the manufacturer's instructions (Miltenyi Biotec, USA). Macrophage polarization was determined as described previously (26). Briefly, RAW 264.7 cells were reseeded in 12-well plates and then stimulated with LPS (1 μg/ml)/IFN-γ (20 ng/ml) for 4 h to induce M1 phenotype; M2 phenotype cells were generated by using IL-4 (20 ng/ml) on RAW 264.7 cells for 4 h. The cells were, respectively, pretreated with 30/60/120 nmol/L of PZQ for 24 h before the induction of the M1 or M2 phenotype. Oligonucleotide sequences of mimic miR-21 and mimic-NC (negative control oligonucleotides) synthesized by Nanjing Qingke Co., Ltd. (Nanjing, China), were cloned into the pEGFP-C1 vector (Invitrogen, USA). For miR-21 overexpression studies in cells, Lipofectamine 2000 (Invitrogen, USA) was employed to transfect the plasmids according to the instruction of the manufacturer. Briefly, RAW24 cells were seeded in 48-well cell culture plates. The next day, cells were transfected with diluted pEGFP-miR-21or pEGFP-NC in Opti-MEM with diluted Lipofectamine 2000. Forty-eight hours later, the fluorescence intensity was observed under a fluorescence microscope.

### Quantitative Real-Time Polymerase Chain Reaction

Total RNA was extracted by using TRIzol reagent (Invitrogen, USA). The concentration of RNA was determined by a NanoDrop2000 spectrophotometer and converted into cDNA with PrimeScript RT Master Mix (Invitrogen, USA). Primers were designed with Primer5.0 software according to the gene sequence in the GenBank database and synthesized by Nanjing Qinke Co., Ltd. The reaction system for miRNA quantitative real-time polymerase chain reaction (qPCR) was designed according to the instructions of a One Step SYBR® PrimeScript® PLUS RT-PCR Kit (Takara, Japan). miRNA expression was normalized using the 2^−ΔΔCt^ method from the Ct values relative to the housekeeping gene U6. Primer sequences are listed in Supplementary Table 1.

### Western Blot Analysis

Total proteins from RAW 264.7 cells were extracted using RIPA lysis buffer containing a protease inhibitor cocktail. Proteins extracted were boiled for 5 min with loading buffer and then loaded on 12% SDS-PAGE running for 1.5 h at 120 V. After being transferred onto the PVDF membrane and being blocked using 5% non-fat milk in Tris-buffered saline with 0.1% Tween-20 (TBST), the membranes were incubated with primary antibodies for smad7 and β-actin (1:1,000; Cell Signaling, USA) overnight at 4°C. The goat anti-rabbit IgG HRP-conjugated antibody was used as a secondary antibody. Bands corresponding to the smad7 protein of the different groups were accessed relative to the normal control group after normalization to β-actin using Image J Software.

### Statistics

The statistical analyses were performed with SPSS version 19.0 software. Two-tailed unpaired Student's *t-*test was used for comparison between two groups. One-way ANOVA followed by Tukey posttest was used for comparison among groups within more than two groups. All experiments were repeated at least three times. The data were represented as the mean ± SEM. *P* < 0.05 was considered statistically significant.

## Results

### Expression of Inflammatory Factors in Splenic Macrophages of Chronic *S. japonicum* Infection

**Figure 1:**
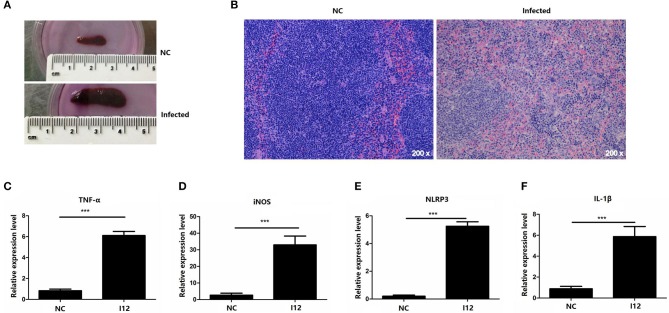
The expression of inflammatory factors in splenic macrophages isolated from chronic *Schistosoma japonicum*–infected mice. **(A)** Gross examination of the spleens from *S. japonicum*-infected mice and normal control mice. **(B)** Representative images of spleens from hematoxylin–eosin (H&E) staining. The mRNA levels of TNF-α **(C)**, iNOS **(D)**, NLRP3 **(E)**, and IL-1β **(F)** in splenic macrophages from mice infected with *S. japonicum* for 12 weeks (I12) and normal control (NC) group without infection were evaluated by quantitative real-time polymerase chain reaction (qPCR). Data are presented as mean ± SD and at least three separate experiments in all studies. ***p < 0.001.

In the chronic *S. japonicum*-infected mouse model, the sizes of spleens from mice with *S. japonicum* infection were significantly increased compared with normal control mice (Figure 1A). The histological structure of the spleens was disorganized, with a disordered distribution of red and white pulp, as well as cell arrangement in infected mice, shown by H&E staining (Figure 1B). In order to explore the differentiation and function of splenic macrophages in the chronic stage of *S. japonicum* infection, we examined the expression of specific genes including iNOS, TNF-α, and inflammasome-related genes NLRP3 and IL-1β. The results showed that in the chronic phase of *S. japonicum* infection, M1-related genes iNOS and TNF-α were more highly expressed in splenic macrophages than in the normal control group (Figures 1C,D). Moreover, the expression of inflammasome-related genes NLRP3 and IL-1β was elevated in infected splenic macrophages (Figures 1E,F). These results suggested that splenic macrophages presented the M1-type and may promote inflammation by regulating NLRP3 inflammasome in the chronic stage of *S. japonicum* infection.

### PZQ Significantly Suppressed Activation of NLRP3 Inflammasome in M1 Macrophages

**Figure 2:**
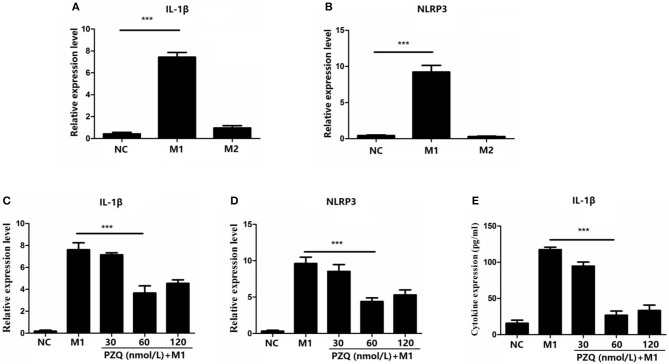
Effect of different concentrations of praziquantel (PZQ) on the activation of NLRP3 inflammasome in M1 macrophages. RAW 264.7 macrophages were induced to M1-type or M2-type, respectively. Fold change in the levels of IL-1β **(A)** and NLRP3 **(B)** mRNA in M1-type and M2-type macrophages measured by qPCR. The effect of different concentrations of PZQ (30, 60, 120 nmol/L) on the gene expression of IL-1β **(C)** and NLRP3 **(D)** in M1-type macrophages measured by qPCR. **(E)** Effect of different concentrations of PZQ on IL-1β levels in the supernatants of cultured M1 macrophages determined by enzyme-linked immunosorbent assay (ELISA). ***p < 0.001.

To assess the effect of PZQ on activation of NLRP3 inflammasome in macrophages, lipopolysaccharide (LPS)/gamma interferon (IFN-γ) or IL-4 was used to stimulate the murine macrophage line RAW 264.7, which was the classical method for inducing macrophage to M1 or M2 phenotype *in vitro* (Supplementary Figure 1). As Figures 2A,B show, M1 macrophages showed higher expression of NLRP3 and IL-1β compared with M2 macrophages. PZQ had no effect on the expression of the IL-1β and NLRP3 in normal RAW 264.7 cells (Supplementary Figure 2). However, the expression of IL-1β and NLRP3 in M1 macrophages was conspicuously suppressed by different concentrations of PZQ pretreatment (Figures 2C,D). Moreover, the secretion level of IL-1β coincided with the detection on the gene level (Figure 2E). And the decreased effect of PZQ on NLRP3 inflammasome activation was shown in the bone marrow–derived macrophages (BMDMs) (Supplementary Figures 3A–F). All the above results indicated that PZQ suppressed the NLRP3 inflammasome activation in M1 macrophages.

### MiR-21 Is Involved in PZQ Inhibiting NLRP3 Inflammasome in M1 Macrophages

**Figure 3:**
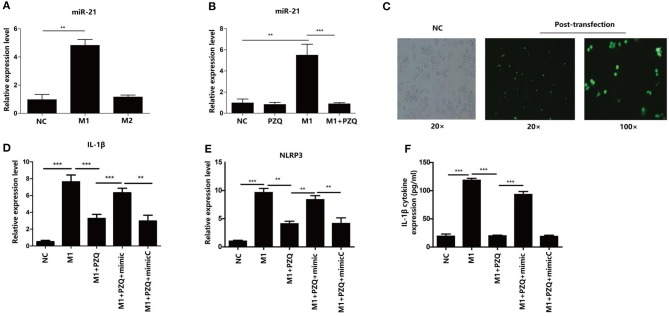
MicroRNA (miR-21) mediated PZQ inhibiting NLRP3 inflammasome activation. **(A)** Comparison of miR-21 expression detected by qPCR in polarized macrophage. **(B)** The effect of PZQ on the expression of miR-21 in M1 macrophages was assessed by qPCR. **p < 0.01 and ***p < 0.001 compared with control. **(C)** The transfection efficiency of miR-21 mimic in RAW264.7 macrophages was observed using a fluorescence microscope. **(D,E)** Macrophages were transfected with miR-21 mimic or the scrambled control; the levels of IL-1β and NLRP3 mRNA in macrophages from the different groups were evaluated by qPCR. **(F)** Secretion of IL-1β in separate groups was detected by ELISA. NC, normal control; M1+PZQ+mimic, M1-type macrophages transfected with miR-21 mimic with PZQ pretreatment; M1+PZQ+mimicC, M1-type macrophages transfected with miR-21 mimic scrambled control with PZQ pretreatment. **p < 0.01, and ***p < 0.001.

To explore the mechanism of the effect of PZQ on the activation of NLRP3 inflammasome in M1 macrophages, the expressions of miR-21 in polarized macrophages were detected by qPCR. As Figure 3A shows, miR-21 expression was higher in M1 macrophages than M2 macrophages. In the presence of PZQ, the expression of miR-21 was considerably decreased in both M1 RAW 264.7 macrophages (Figure 3B) and M1 BMDMs (Supplementary Figure 3H). These results indicated that PZQ might regulate the inflammatory response by inhibiting the expression of miR-21. To further confirm the role of miR-21 in anti-inflammation effects of PZQ, we transfected miR-21 mimic into M1 RAW 264.7 macrophages and detected the activation of NLRP3 inflammasome with or without PZQ pretreatment. The expression of green fluorescent protein in the transfected cells was observed under a fluorescence microscope (Figure 3C). The results of qPCR and ELISA showed that miR-21 overexpression could abolish the PZQ-induced inhibition of the NLRP3 and IL-1β genes (Figures 3D,E) as well as the secretion of IL-1β (Figure 3F) in M1 macrophages.

### MiR-21 Targets Smad7 to Mediate Anti-inflammation of PZQ

**Figure 4:**
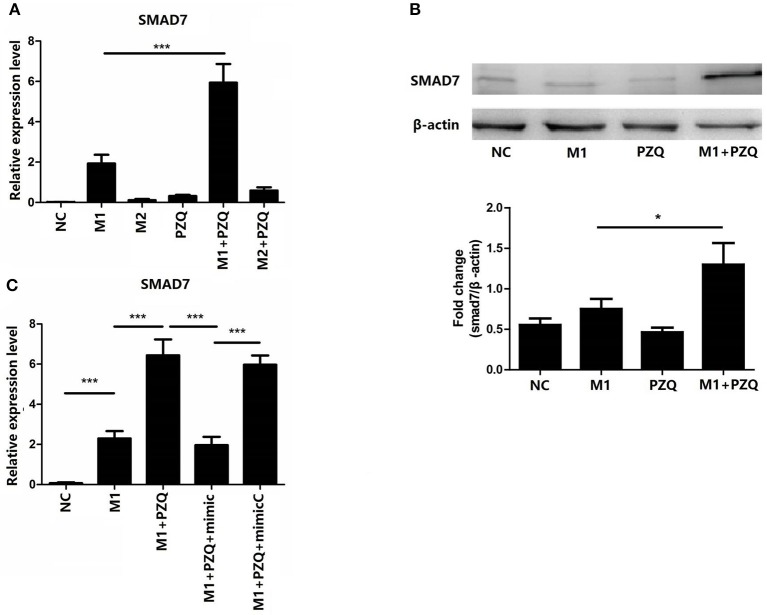
Overexpression of miR-21 abolished PZQ-induced Smad7 increase in M1 macrophages. The gene **(A)** and protein **(B)** levels of Smad7 in macrophages from the different groups were evaluated by qPCR or western blot, respectively. **(C)** The effect of miR-21 overexpression on Smad7 mRNA level in the presence of PZQ. *p < 0.05 and ***p < 0.001.

A recent study has shown that miR-21 mediates NLRP3 inflammasome via targeting Smad7 (23). To elucidate whether Smad7 was involved in the inhibition effect of PZQ on NLRP3 inflammasome, the gene and protein expression of Smad7 in polarized macrophages with or without PZQ treatment was measured. Smad7 expression was remarkably increased in the presence of PZQ in M1 macrophages (Figures 4A,B and Supplementary Figure 3G). Moreover, Smad7 was measured in PZQ-pretreated M1 macrophages with miR-21 overexpression. The results showed that the expression of Smad7 was decreased in the miR-21 mimic group with PZQ treatment, compared to the PZQ treatment alone group as well as the mimic-normal control group (Figure 4C).

## Discussion

Hepatic egg granuloma/fibrosis and splenomegaly/ hypersplenism are the main pathological damages caused by *S. japonicum* infection. There is still no effective treatment for the pathological damage of the spleen in patients with schistosomiasis, besides total or partial resection of the spleen. Therefore, it is necessary to study the pathogenic mechanism of spleen pathological damage caused by schistosomiasis for clinical treatment.

Inflammatory response plays an important role in the pathological damage such as liver fibrosis, intestinal inflammation, and splenomegaly during schistosomiasis. Macrophages, as one kind of important immunoregulatory cell, play an irreplaceable role in the process of inflammatory reaction as well as the pathological damage process of the spleen (27). Therefore, the in-depth study of macrophage-related inflammation is extremely important to clarify the pathogenesis of schistosomiasis characterized by splenomegaly.

Macrophages display different biological functions depending on their polarization state (28). Classically activated macrophages M1 have generally pro-inflammatory properties, while activated macrophages M2 have an anti-inflammatory effect with downregulated immune responses (29). The polarization of macrophages is largely influenced by their microenvironment (30). It has been reported that liver and peritoneal macrophages are mainly M1 macrophages in the early stage and then polarize to M2 macrophages in the chronic phase during *S. japonicum* infection (31, 32). However, the present study showed that M1-type was the main splenic macrophage in the chronic infection phase of schistosomiasis, indicating that the immune environment and status of different organs may be different during *S. japonicum* infection. Note that the splenic macrophages in this study were sorted by CD11b magnetic bead, and anti-F4/80 and anti-CD11b antibodies were used to further identify the purity of sorted cells (Supplementary Figure 4). It is reported that F4/80 is the dominant marker of splenic macrophages (33); our sorting strategy of splenic macrophages by using CD11b magnetic beads may disregard F4/80^+^ CD11b^−^ macrophages. However, this cannot negate our study on the role of macrophages in the splenic pathogenesis of *S. japonicum* infection.

NLRP3 inflammasome is a multiprotein complex formed in the cytoplasm during infection to cope with cell damage. So NLRP3 is recognized as an important mediator of aseptic inflammation and autoimmune diseases (34). Splenic macrophages highly expressed NLRP3 inflammasome–related factors in the chronic phase of *S. japonicum* infection. Meanwhile, activation of NLRP3 inflammasome in M1 macrophages was also demonstrated in macrophage cell line RAW264.7 polarization *in vitro*. Therefore, M1 macrophages play an important role in the process of inflammatory reaction by activating NLRP3 inflammasome.

miRs are endogenous and highly conserved single-stranded non-coding RNAs (35, 36). A variety of miRs have been reported to be involved in the regulation of NLRP3 inflammasome (37, 38). Among miRs, it is reported that miR-21 is particularly involved in inflammatory reaction (39). The present study showed that NLRP3 inflammasome was highly expressed in M1 macrophages. This together with the expression of miR-21 serve as clues that macrophages might show a pro-inflammatory phenotype via miR-21.

**Figure 5:**
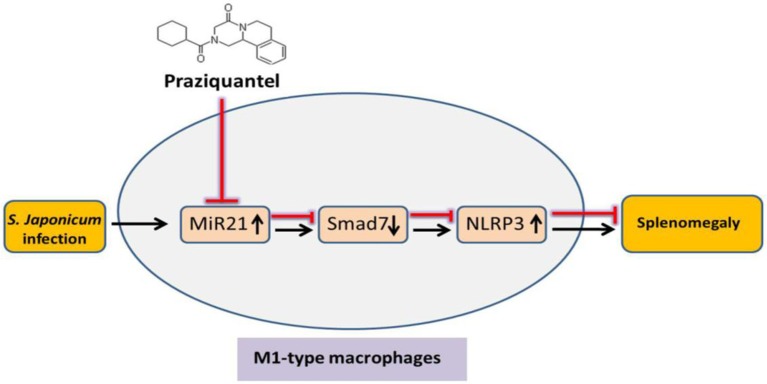
Schematic diagram of the inhibiting effect of PZQ on NLRP3 inflammasome in M1-type macrophages via miR-21. PZQ increased the expression of Smad7 by downregulating miR-21, thereby inhibiting the activation of NLRP3 inflammasome in M1-type macrophages. This can further result in the anti-inflammatory effect of PZQ and considerably improved *S. japonicum*-induced splenomegaly.

Studies have suggested that PZQ not only has an anti-parasitic effect but also plays an important role in regulating the function of immune cells (40, 41). PZQ could downregulate the expression of inflammatory factors and chemokines in a mouse model of *S. japonicum* infection and a ConA-induced hepatitis (16). Studies found that depressive-like behaviors depended on the activation of NLRP3 inflammasome. The anti-depressant fluoxetine alleviates NLRP3 inflammasome by activating 5HT2B receptor (42). Meanwhile, the eutomer of PZQ has been shown to be a partial agonist of the human 5HT2B receptor (43). These data as well as our results indicate that PZQ could inhibit NLRP3 inflammasome activation. It is reported that the high level of miR-21 induced by *Opisthorchis viverrini*-infection in patients was reduced significantly by PZQ treatment (44). Moreover, PZQ treatment significantly reduced spleen pathological damage induced by chronic infection of schistosomiasis in mice (18). A recent study has shown that PZQ promotes human Type 1 regulatory T cell differentiation, suggesting that PZQ may also have immunomodulatory functions in parasite-unrelated human inflammatory diseases (45). Given that evidence, the anti-inflammatory effect of PZQ might be benefit from its inhibition of the NLRP3 inflammasome signaling pathway in M1 macrophages and its reduction of miR-21 expression. Therefore, we speculated that the anti-inflammatory effect of PZQ was caused by the interaction of PZQ, miR-21, and NLRP3. To clarify the relationship between them, miR-21 was overexpressed in macrophages after being pretreated with PZQ. Our data showed that the expression of NLRP3 inflammasome signaling pathway–associated factors was re-elevated. Therefore, PZQ suppressed the activation of NLRP3 by regulating the expression of miR-21. Given the general negatively regulating effect of miRs, it is supposed that certain molecules are involved in the regulation of miR-21 on NLRP3. It is reported that Smad7 is a target of miR-21 (46). Recent research also reported that miR-21 activated NLRP3 inflammasome via targeting Smad7 (23). In this study, PZQ promoted Smad7 expression in macrophages, while miR-21 overexpression abrogated this effect. However, the underlying regulatory mechanism of activation of NLRP3 inflammasome by Smad7, as well as the function of Smad7 in splenic macrophages during *S. japonicum* infection, remains unclear. The present study indicated that PZQ might indirectly mediate the expression of Smad7 by inhibiting miR-21, thereby inhibiting the activation of NLRP3 inflammasome (Figure 5). Nevertheless, it is necessary to conduct further studies *in vivo* to demonstrate the relationship between them in detail.

## Conclusion

In this study, we confirmed that M1 is the predominant type of splenic macrophage in mice with chronic schistosomiasis infection and the NLRP3 inflammasome signaling pathway–related molecules were upregulated in infected macrophages. Furthermore, miR-21 mediated the suppression effect of PZQ on the NLRP3 inflammasome signaling pathway via targeting Smad7. The present study hopefully stimulates further investigations on the mechanism of the PZQ effect on splenic damage induced by schistosomiasis infection.

## Data Availability Statement

All datasets generated for this study are included in the article/Supplementary Material.

## Ethics Statement

The animal study was reviewed and approved by Institutional Animal Care and Use Committee (IACUC) of Nanjing Medical University (IACUC no. 1601159).

## Author Contributions

DK conceived and designed the experiments. ZL and HG performed the experiments. DK and JC analyzed the data and wrote the paper. All authors read and approved the final version of the manuscript.

### Conflict of Interest

The authors declare that the research was conducted in the absence of any commercial or financial relationships that could be construed as a potential conflict of interest.
