# Supplementary material for: MicroRNA-21 Mediates the Inhibiting Effect of Praziquantel on NLRP3 Inflammasome in Schistosoma japonicum Infection
Source: Front Vet Sci. 2020 Feb 12;6:517. doi: 10.3389/fvets.2019.00517 (PMC7029728; doi:10.3389/fvets.2019.00517)
Supplement: Supplementary file 1 [file Data_Sheet_1.PDF]

Supplementary table 1 The primer pairs used in this study (5'-3')

Name		Sequence（**5′→3′**）
GAPDH	Forward primer	AGGTCGGTGTGAACGGATTTG
	Reverse primer	TGTAGACCATGTAGTTGAGGTCA
Arg-1	Forward primer	CTCCAAGCCAAAGTCCTTAGAG
	Reverse primer	AGGAGCTGTCATTAGGGACATC
iNOS	Forward primer	GTTCTCAGCCCAACAATACAAGA
	Reverse primer	GTGGACGGGTCGATGTCAC
TNF-α	Forward primer	ATAGCTCCCAGAAAAGCAAGC
	Reverse primer	TTGGTCCTTAGCCACTCCTTC
Smad7	Forward primer	GGCCGGATCTCAGGCATTC
	Reverse primer	TTGGGTATCTGGATAAGGAGG
NLRP3	Forward primer	GCUUCAGCCACAUGACUUU
	Reverse primer	AAAGUCAUGUGGCUGAAGC
IL-1β	Forward primer	CCTCGTGCTGTCGGACCCATA
	Reverse primer	CAGGCTTGTGCTCTGCTTGTGA
MiR-21	Forward primer	GCACCGTCAAGGCTGAGAAC
	Reverse primer	CAGCCCATCGACTGGTG
U6	Forward primer	CTCGCTTCGGCAGCACA
	Reverse primer	AACGCTTCACGAATTTGCGT

SUPPLEMENTARY FIGURE LEGENDS

Supplementary Fig. 1 RAW 264.7 macrophages were induced to M1 or M2 phenotype. Lipopolysaccharide (LPS)/gamma interferon (IFN-γ) or IL-4 was used to induce the murine macrophage line RAW 264.7 to the M1 or M2 phenotype, the markers of the M1 phenotype (TNF-α, iNOS) and M2 phenotype marker (Arg-1) were detected by qPCR. ***, $p < 0.001$.

Supplementary Fig. 2 PZQ had no effect on NLRP3 and IL-1β gene expression in normal macrophages. RAW 264.7 cells were reseeded in 24-well plates with or without different concentrations of PZQ treatment for 24 hours. The effect of PZQ treatment alone on NLRP3 and IL-1β gene expression in macrophages were detected by q-PCR.

Supplementary Fig. 3 PZQ inhibited the activation of NLRP3 inflammasome in induced M1-type bone marrow derived macrophages. Femur and tibia bones are collected from 6-8 weeks mice and bone marrow cells flushed out using PBS supplemented with 2% heat inactivated FBS. After red blood cells are lysed with NH4Cl solution, cells are cultured in BMDM growth medium (Iscove's Modified Dulbecco Medium + 10% FBS + 10 ng/ml M-CSF) for 7 days followed by maturation and FCM analysis for purity staining with anti-F4/80 and anti-CD11b antibodies (Fig. 4 A). Polarized macrophages can be evaluated on the basis of changes in cell morphology (Fig. 4 B), surface marker (M1: TNF-α/iNOS and M2: Arg-1) presentation (Fig.4 C). and then the effects of PZQ on the levels of IL-1β (Fig. 4 D), NLRP3 (Fig. 4 E), Smad7 (Fig. 4 G) and miR-21 (Fig. 4 H) mRNA in M1-type BMDMs determined by quantitative PCR as well as IL-1β protein levels in the supernatants of cultured cells detected by ELISA (Fig. 4 F). *, $p < 0.05$, **, $p < 0.01$ and ***, $p < 0.001$.

Supplementary Fig. 4 The purity of isolated splenic macrophages was

determined by flow cytometry. The isolated splenic macrophages were stained with PE-conjugated anti-F4/80 and PercP-cy5.5-conjugated anti-CD11b antibodies. Expression of F4/80 and CD11b in sorted cells were evaluated by flow cytometry analysis.
