# Supplementary figures and images for: MicroRNA-21 Mediates the Inhibiting Effect of Praziquantel on NLRP3 Inflammasome in Schistosoma japonicum Infection
Source: Front Vet Sci. 2020 Feb 12;6:517. doi: 10.3389/fvets.2019.00517 (PMC7029728; doi:10.3389/fvets.2019.00517)

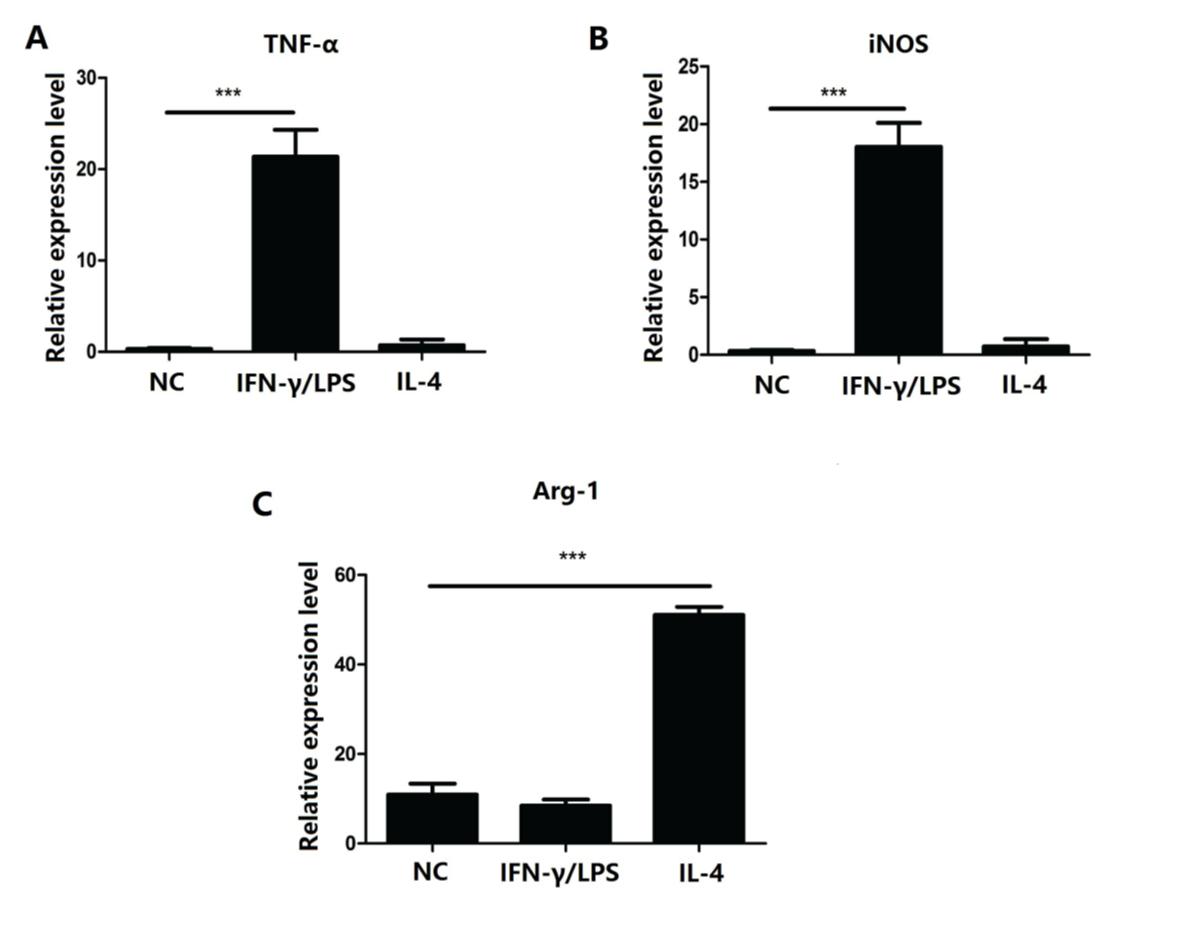

Supplement: Supplementary file 2 [file Image_1.JPEG]

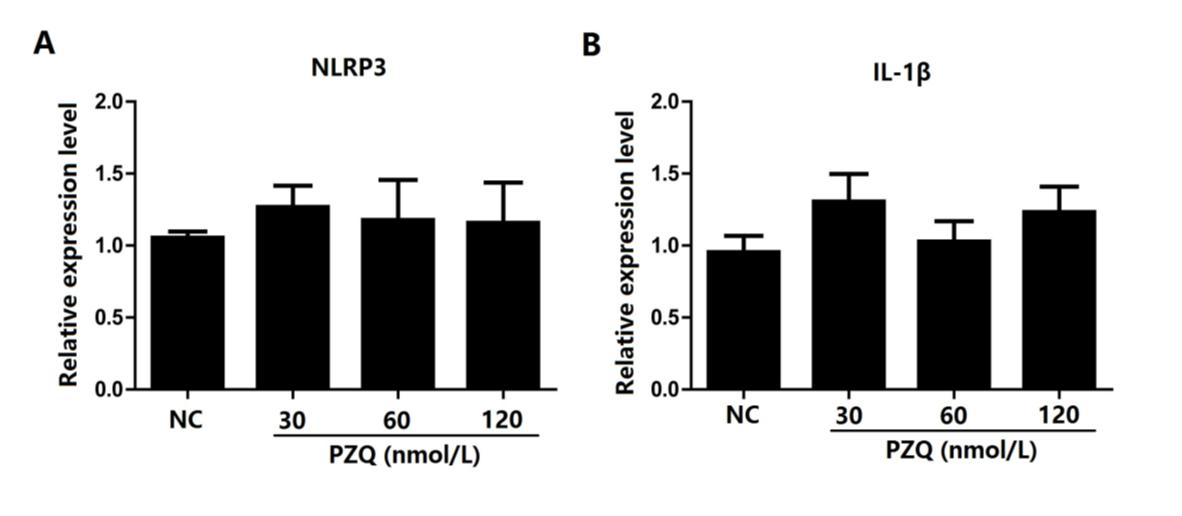

Supplement: Supplementary file 3 [file Image_2.JPEG]

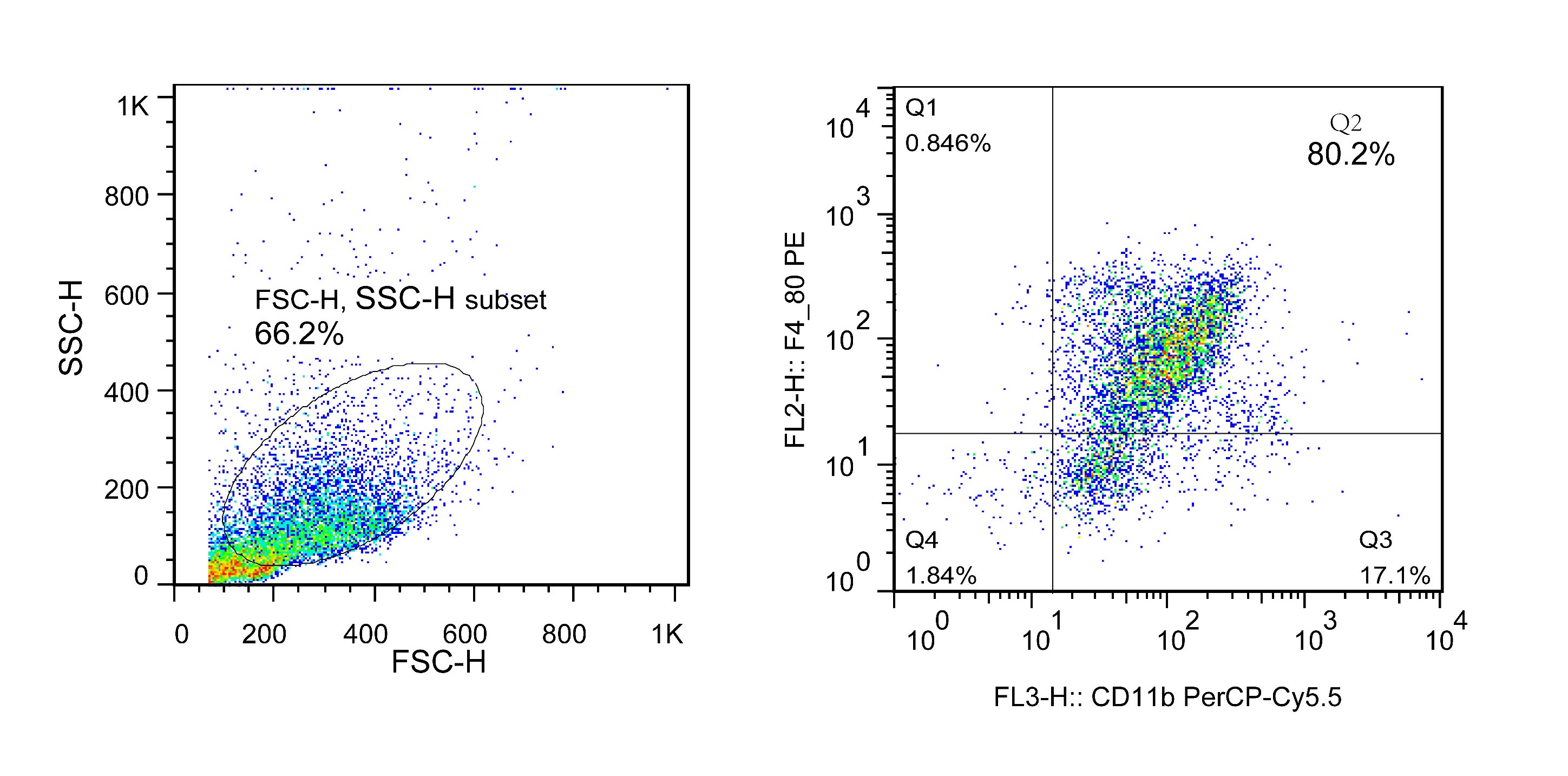

Supplement: Supplementary file 5 [file Image_4.JPEG]
